# rEHR: An R package for manipulating and analysing Electronic Health Record data

**DOI:** 10.1371/journal.pone.0171784

**Published:** 2017-02-23

**Authors:** David A. Springate, Rosa Parisi, Ivan Olier, David Reeves, Evangelos Kontopantelis

**Affiliations:** 1 NIHR School for Primary Care Research, University of Manchester, Manchester, United Kingdom; 2 Centre for Biostatistics, Faculty of Biology, Medicine & Health, University of Manchester, Manchester, United Kingdom; 3 Centre for Pharmacoepidemiology & Drug Safety, Faculty of Biology, Medicine & Health, University of Manchester, Manchester, United Kingdom; 4 Informatics Research Centre, School of Computing Mathematics and Digital Technology, Manchester Metropolitan University, Manchester, United Kingdom; 5 The Farr Institute for Health Informatics Research, Faculty of Biology, Medicine & Health, University of Manchester, Manchester, United Kingdom; Indiana University, UNITED STATES

## Abstract

Research with structured Electronic Health Records (EHRs) is expanding as data becomes more accessible; analytic methods advance; and the scientific validity of such studies is increasingly accepted. However, data science methodology to enable the rapid searching/extraction, cleaning and analysis of these large, often complex, datasets is less well developed. In addition, commonly used software is inadequate, resulting in bottlenecks in research workflows and in obstacles to increased transparency and reproducibility of the research. Preparing a research-ready dataset from EHRs is a complex and time consuming task requiring substantial data science skills, even for simple designs. In addition, certain aspects of the workflow are computationally intensive, for example extraction of longitudinal data and matching controls to a large cohort, which may take days or even weeks to run using standard software. The rEHR package simplifies and accelerates the process of extracting ready-for-analysis datasets from EHR databases. It has a simple import function to a database backend that greatly accelerates data access times. A set of generic query functions allow users to extract data efficiently without needing detailed knowledge of SQL queries. Longitudinal data extractions can also be made in a single command, making use of parallel processing. The package also contains functions for cutting data by time-varying covariates, matching controls to cases, unit conversion and construction of clinical code lists. There are also functions to synthesise dummy EHR. The package has been tested with one for the largest primary care EHRs, the Clinical Practice Research Datalink (CPRD), but allows for a common interface to other EHRs. This simplified and accelerated work flow for EHR data extraction results in simpler, cleaner scripts that are more easily debugged, shared and reproduced.

## 1 Introduction

We present the R R [1] package rEHR for manipulating and analysing Electronic Health Record (EHR) data and demonstrate its use with rEHR-generated synthetic data. rEHR is available from the Comprehensive R Archive Network (CRAN) at https://CRAN.R-project.org/package=rEHR, and will work with R-3.3.2.

The package has been developed using structured primary care data from the UK, which has enjoyed near-universal deployment of EHRs in general practice and clinical coding performed by general practitioners for over twenty years. Comprehensive anonymised extracts of these UK primary care records are made available for research—the main sources are: The Clinical Practice Research Datalink (CPRD, previously known as the General Practice Research Database, GPRD), The Health Improvement Network (THIN), QResearch, The Doctors’ Independent Network (DIN-LINK) and more recently, Research One. These databases hold near complete anonymised medical records for millions of patients and have very similar structures, including data on demographics, symptoms, tests, diagnoses, therapies, health-related behaviours and complete referrals to secondary care (since in the UK general practitioners are the gatekeepers of the health care system and manage specialist referrals). Using the CPRD as an example, in 2015 it reported coverage for over 1.3 million patients from 674 UK practices, with 4.4 million active (alive, meeting CPRD quality criteria and registered with one of the general practices at the end of 2015) patients thus covering approximately 6.9% of the UK population [2]. Due to the fact that these databases tend to be tied to specific clinical systems which demonstrate regional variability [3], there are some UK regions that are under-represented in each [4]. However, the size of all these databases ensures that included patients are broadly representative of the UK general population in terms of age, sex and ethnicity. To date, over 1600 papers have been published using these UK primary care databases (PCDs), with well over 150 papers published per year since 2012. EHR research is set to grow still faster due to advances in analysis methodology [5; 6], an increasing body of evidence supporting the validity of such studies [7; 8], and efforts to improve transparency and reproducibility [9].

Despite the research interest in PCDs, data science methodology to enable the rapid searching/extraction, cleaning and analysis of these increasingly large and complex datasets is less well developed. In addition, commonly used software tools are often inadequate, resulting in bottlenecks in the research workflow and in obstacles to increased transparency and reproducibility of research. PCDs such as CPRD store data in complex relational and nested structures, and preparing an analysis-ready dataset requires substantial data science skills, even for simple designs. This complexity is an inevitable consequence of the wide range of information contained within these databases, which detail the primary care history for every patient, including coded data for all diagnoses, prescriptions, referrals and test results for all consultations. To manage this vast wealth of data requires a relational structure based on multiple tables, classifications and terminologies (e.g. Read codes for diagnoses and referrals, product codes for prescriptions). To extract relevant data, research teams have to complete a sequence of non-trivial technical tasks. The more complex the research design the more steps are required to obtain the final dataset. For example, investigating drug outcomes typically involves constructing complex definitions of codes for diagnosis, drug exposure (may be varying over time), mortality, and possible confounding factors (e.g. comorbidities, additional medications, gender, age, referrals, date of diagnosis, etc.). In addition, certain aspects of the workflow are computationally intensive (for example extraction of longitudinal data and matching controls to a large cohort)—often taking days or even weeks to run using standard software. Although more powerful computer facilities help (and are practically a prerequisite for working with these data), an inefficient and slow program running on a fast server will still be inefficient and slow. Some ‘how-to’ papers exist for good practice in observational data management but they address only some of the issues or focus on specific applications [5; 10; 11; 12]. At the same time there is a wealth of health informatics and computer science literature on how to make these research processes more transparent, reducing the duplication of effort and improving the consistency of data processing [13; 14]. Finally, several software packages exist for speeding up data analysis, but these are generic, do not apply directly to EHR manipulation and may require specialist knowledge to effectively use for fast manipulation of dataframes [15], for database integration [16], and for parallel processing (parallel in base R).

rEHR simplifies and accelerates the process of extracting ready-for-analysis datasets from EHR databases. In section 2 we provide instructions on loading the software and importing flat text files of the kind supplied by EHR providers into a local SQL database. In section 3 we describe the basic query operations provided by the package, the building of longitudinal data and calculation of prevalence and incidence statistics. In section 4 we convert the longitudinal data from the previous section to a cohort dataset suitable for survival analysis and illustrate algorithms to match controls to cases and to cut cohort data by time-varying covariates. In section 5 we briefly discuss some accessory functions provided in the package. In the final section we discuss the .ehr environment used to define the EHR database being used and how this can be set to work with different databases.

The package includes a number of simulated flat files to allow users to familiarise themselves with advanced aspects, which we use in this paper to provide examples.

## 2 Importing EHR data

rEHR is installed and loaded in the usual way:

if(! "rEHR" %in% rownames(installed.packages())) install.packages("rEHR")

library(rEHR)

The development version of the package is available from Github and is accessible via the devtools package [17]:

library(devtools)

install_github("rOpenHealth/rEHR")

library(rEHR)

EHR data are stored as relational databases but are most commonly made available to researchers in the form of flat text files. This has the advantage of easier access for simple tasks and, for example, viewing the files in a spreadsheet. However, most non-trivial operations require researchers to iterate over a series of (potentially large) different groups of files. For example here we present pseudocode for a simple workflow leading to the production of a dataset of prevalent cases for a condition such as diabetes:

#Pseudocode prevalent cases algorithm

define a list of clinical codes for the condition

for each practice:

 load clinical events files (clinical, referral, drugs etc.)

 select clinical events matching the clinical code list

 load patient and practice files

 for each year:

  select active patients

  select events in year

  merge active patients and events in year on condition algorithm

 combine all years in practice

combine patients in all practices

Each level of iteration (represented by the nested for loops) and each type of file (e.g. clinical, referral, drugs etc.) in the above algorithm introduces the opportunity for bugs to creep into extraction code, while the repeated opening and closing of multiple text files, combined with the inherent inefficiency of for loops in R often result in slow, error prone code. The rEHR package allows researchers to first automatically import these flat files into a SQLite database and then use predefined functions to query this database efficiently and precisely. We use SQLite databases for a variety of reasons:

- SQLite databases are stored as files in the directory system of the computer and require no installation setup. SQLite3 is installed automatically as a result of installing the dependencies for the package
- SQLite files are stored efficiently and are relatively small compared to text files
- The SQL language has been optimised for very rapid and efficient queries of SQLite files, resulting in much faster queries than would be available to multiple flat files
- Working with SQLite databases allows users to use some very well developed tools that are already available to the R community such as sqldf [16] and RSQLite [18] if they are familiar with R SQL integration tools. These tools also allow for more specific tool functions to be built to shield users from the complexities of SQL queries.

## Use simulated ehr files supplied with the package to build database

ehr_path <- dirname(system.file("ehr_data", "ehr_Clinical.txt",

    package = "rEHR"))

## create a new database connection to a temporary file

db <- database(tempfile(fileext = ".sqlite"))

## Import multiple data files into the database

import_CPRD_data(db, data_dir = ehr_path,

         filetypes = c("Clinical", "Consultation",

                 "Patient", "Practice",

                 "Referral"),

         dateformat = "%Y‐%m‐%d",

         yob_origin = 1800,

         regex = "ehr",

         recursive = TRUE)

## Individual files can also be added:

add_to_database(db, files = system.file("ehr_data", "ehr_Therapy.txt",

    package = "rEHR"),

        table_name = "Therapy", dateformat = "%Y‐%m‐%d")

## Use the overloaded `head` function to view a list of

## tables or the head of individual tables:

head(db)

##  type   name   tbl_name

## 1 table   Clinical   Clinical

## 2 table Consultation Consultation

## 3 table    Patient    Patient

## 4 table   Practice   Practice

## 5 table   Referral   Referral

## 6 table   Therapy   Therapy

head(db, table = "Clinical")

##  patid  eventdate constype consid medcode   comorbidity practid

## 1 1001 2003-08-25     0    4   69753   hypertension   1

## 2 1001 2004-04-13     1    5   96277  atrial_fibrilation    1

## 3 1001 2004-04-13     1    5   2212 atrial_fibrilation    1

## 4 1001 2004-04-13     1    5   96076  atrial_fibrilation    1

## 5 1001 2005-02-08     1    6   23579       chd    1

## 6 1001 2005-02-18     1    7   16059   hypertension    1

The import_CPRD_data and add_to_database functions are able to import tab-delimited text files or zipped tab-delimited text-files. By default, all date strings are converted to R dates with standard ISO format (“%Y-%m-%d”). A regex argument should be supplied that is a regular expression to match a common prefix to the filenames, separated from the file type by an underscore.

## 3 Querying the database

### 3.1 Selecting all events

Once EHR data has been imported to the database, the rEHR package has a number of flexible built-in querying functions for extracting data. These functions are much faster to execute and less error prone than having to loop through hundreds of text files.

The primary generic query function is select_events() and is able to select all the events in a database table matching a provided where argument. This function is also called by the other more specific query functions. An example set of lists of clinical codes for a number of medical conditions is provided with the package (data(clinical_codes)). select_events() returns a dataframe of extracted data. This collection of disease specific code lists stems from our previous work and are reposited in www.clinicalcodes.org [9]. However, code lists are dynamic and context specific and researchers will very likely need to consider strategies to develop their own code lists, if existing code lists are considered inadequate [19].

diabetes_codes <- clinical_codes[clinical_codes$list == "Diabetes",]

select_events(db, tab = "Clinical", columns = c("patid", "eventdate",

      "medcode"),

      where = "medcode %in%.(diabetes_codes$medcode) &

              eventdate<'2006‐01‐01'&eventdate>='2005‐01‐01'")

##  patid  eventdate medcode

## 1 3012 2005-09-30    273

## 2 1037 2005-04-08    277

## 3 1038 2005-05-19    273

## 4 1091 2005-05-27    351

## 5 1091 2005-07-25    351

## 6 1097 2005-03-10    273

The tab argument is used to select the file type (Clinical, Consultation, Patient, Practice or Referral in the previous code example), while the columns argument selects variables from these files. The where argument is equivalent to the WHERE clause in SQL, in that it is used to select subsets of the data table. The user must supply a string representation of valid R code, which is then translated to SQL via the dplyr::translate_sql_ function. There are two important caveats to this:

1. If an element of the clause represents an R object to be accessed (such as the elements of a vector) it must be wrapped in a .() (See the example above). String elements wrapped in .() are processed by the expand_string function before being passed to dplyr::translate_sql_.
2. Dates should separately quoted and entered in ISO format (‘%Y-%m-%d’). This is because dates are stored as ISO text in the database, not as r Date types.

If the argument sql_only == TRUE, the function only generates the SQL needed for the query, rather than running the query itself. In this way, select_events can be used as the base for more complex query functions. The results of this function can also then be passed to temp_table() to create temporary tables where it is not desirable to keep large query results in RAM. For example:

Asthma_codes <- clinical_codes[clinical_codes$list == "Asthma",]

q <- select_events(db, tab = "Clinical", columns = c("patid", "eventdate"

        , "medcode"),

      where = "medcode %in%.(Asthma_codes$medcode)",

      sql_only = TRUE)

temp_table(db, tab_name = "Asthma", select_query = q)

## Temporary table 'Asthma' created

head(db, temp = TRUE)

##   type  name tbl_name

## 1 table Asthma  Asthma

head(db, table = "Asthma")

##  patid  eventdate medcode

## 1  1025 2014-04-11   1105

## 2  1035 2012-03-05   1116

## 3  2065 2006-03-20   1095

#### 3.1.1 Using raw SQL queries

Since EHR data is stored as a standard SQLite database, users can alternatively make SQL queries to the database using sqldf, which is imported into the namespace on loading of the rEHR package:

sqldf("SELECT patid, practid, gender, yob, deathdate from Patient WHERE

     deathdate IS NOT NULL LIMIT 6",

  connection = db)

##  patid practid gender  yob  deathdate

## 1 1003    3   0 1983 2001-11-16

## 2 3015    15   1 1995 2000-05-09

## 3 2016    16   1 1959 2002-10-28

## 4 1018    18   0 1992 2009-12-29

## 5 2020    20   1 1956 2002-11-29

## 6 1023    23   0 1983 2013-03-24

There are two methods for including R objects in raw SQL strings. First, wrapping the string in a call to expand_string() allows for the .() notation to be used as in where arguments to select_events() based functions. Alternatively, a helper function, wrap_sql_query() is provided that functions in a similar way to base::sprintf but formats objects according to SQL syntax. If the result of evaluating the argument is a vector of length 1, it is inserted as is; if it is a vector of length > 1, it is wrapped in parentheses and comma separated.

medcodes1 <- 1:5

practice <- 255

expand_string("SELECT *FROM clinical WHERE practid==.(practice)")

## [1] "SELECT * FROM clinical WHERE practid == 255"

wrap_sql_query("SELECT *FROM clinical WHERE practid==#1 AND medcodes

        in #2", practice, medcodes1)

## [1] "SELECT * FROM clinical WHERE practid == 255 AND medcodes in

(1, 2, 3, 4, 5)"

### 3.2 Selecting first or last events

Frequently, users need to find the first clinical event for a given patient (e.g. to identify dates of diagnosis of chronic diseases) or the most recent clinical event (e.g. to identify if a drug therapy has been prescribed within a certain time period). rEHR provides convenience functions for these common situations. The functions run a select_events() query and then group by patient id and selects only the earliest/latest event for each patient:

first_DM <- first_events(db, tab = "Clinical",

             columns = c("patid", "eventdate", "medcode"),

       where = "medcode %in%.(diabetes_codes$medcode)")

last_DM <- last_events(db, tab = "Clinical",

            columns = c("patid", "eventdate", "medcode"),

       where = "medcode %in%.(diabetes_codes$medcode)")

head(first_DM)

##  patid  eventdate medcode

## 1  1004 2007-12-25    351

## 2  1005 2004-08-31    351

## 3  1008 2002-03-02    351

## 4  1010 2014-04-11    351

## 5  1012 2012-05-28    351

## 6  1015 2008-08-16    351

head(last_DM)

##  patid  eventdate medcode

## 1  1004 2007-12-25    351

## 2  1005 2009-03-09    351

## 3  1008 2002-03-02    351

## 4  1010 2014-04-11    351

## 5  1012 2013-02-14    351

## 6  1015 2013-08-17    273

### 3.3 Querying longitudinal data with select_by_year()

Researchers will often want to extract data over a range of different time-points, for example they may want to calculate the prevalence of a condition and how this changes through time. When working with flat text files, this must be done with a complex nested loop that is both slow and error-prone. The select_by_year() function provides a simple interface to extract longitudinal data. On posix-compliant computers (Linux, BSD, Mac), this function can make use of parallel processes to select data for different years concurrently, greatly accelerating the extraction process on multicore machines. The function runs a series of selects over a year range and collects in a list of dataframes.

The function applies a database select over a range of years and outputs as a list or a dataframe. Either a database object or a path to a database file can be supplied. If multiple cores are being used (i.e. cores > 1), a path to a database file must be used because the same database connection cannot be used across threads. In this case, a new database connection is made with every fork. Note that when working with temporary tables, cores must be set to 1 and the open database connection must be set with db. This is because the use of parallel::mclapply means that new database connections need to be started for each fork and temporary files are only available inside the same connection.

Queries can be made against multiple tables, assuming that the columns being extracted are present in all tables. The columns argument is a character vector of column names to be selected. The individual elements can be of arbitrary length. This means it is possible to insert SQL clauses e.g. “DISTINCT patid”.

A numeric vector of years is passed to the year_range argument to specify the years to select data for. Selection is done according to the function passed to the selector_fn argument. select_events is the default but first_events and last_events can also be used, as well as custom selection functions. The where argument works in the same way as in select_events except that year-start and year-end criteria can be added as ‘STARTDATE’ and ‘ENDDATE’. These are translated to the correct year- start and end dates. Different start and end dates can be specified by supplying a function to the year_fn argument. This function must accept a single year argument and return a list with two elements—“startdate” and “enddate”, each of which must be date characters in posix format (i.e. “%Y-%m-%d”). Three functions are provided to define years (standard_years for 1st January to 31st December, qof_years for UK financial years as used in the UK Quality and Outcomes Framework [20], and qof_15_months for the period starting 1st January in the year in question and finishing on the 31st March the following year) and a convenience function, build_date_fn() is provided to which users can supply lists of year offsets, months and days for year- start and end to return a function that can be supplied as the year_fn argument. Finally the user can set the as_list argument to determine whether data from each year is returned as a separate list element or as a single data frame.

#### 3.3.1 Selecting prevalent and incident events

To show the utility of the package we demonstrate how one might extract an incident and prevalent cohort of diabetes patients from the simulated example data. Prevalent events for a chronic condition are selected by the earliest diagnostic event prior to the end of the time period in question. The denominator for the calculation of the prevalence is the total number of patients registered at that time point.

# Select all patients with current registration date (crd)<the start

# date for each year.

registered_patients <- select_by_year(db = db,

           tables = "patient",

           columns = c("patid", "practid", "gender",

                    "yob", "crd", "tod", "deathdate"),

           where = "crd<STARTDATE",

           year_range = c(2008:2012),

           year_fn= standard_years)

## Using open database connection

str(registered_patients)

## Classes 'tbl_df', 'tbl' and 'data.frame': 1005 obs. of 8 variables:

## $ patid    : int  1001 1002 2002 3002 4002 1003 2003 1004 2004 …

## $ practid   : int  1 2 2 2 2 3 3 4 4 4 …

## $ gender   : int  1 1 1 1 0 0 1 0 1 1 …

## $ yob   : num 1989 1942 1965 1959 1932 …

## $ crd   : chr   "1998-03-22" "2003-07-10" "1997-10-15" …

## $ tod   : chr  NA NA NA NA …

## $ deathdate : chr  NA NA NA NA …

## $ year    : int  2008 2008 2008 2008 2008 2008 2008 2008 2008 …

table(registered_patients$year)

##

## 2008 2009 2010 2011 2012

##  189  195  201  206   214

Notice that select_by_year returns a dataframe in long form, with a year column for the longitudinal component. Next we collect the incident cases, which are those patients with first diagnoses at any point before the end of the year in question, plus the dates for the first diagnoses. In this case we include events matching our list of diabetes clinical codes in either clinical or referral files. Because we only want the first diagnosis dates we set the selector_fn argument to first_events:

incident_cases <- select_by_year(db = db,

                tables = c("Clinical", "Referral"),

                columns = c("patid", "eventdate",

                     "medcode"),

                where = "medcode %in%

                    .(diabetes_codes$medcode)

                    &eventdate<=ENDDATE",

                year_range = c(2008:2012),

                year_fn = standard_years,

                selector_fn= first_events)

## Using open database connection

str(incident_cases)

## Classes 'tbl_df', 'tbl' and 'data.frame': 262 obs. of 5 variables:

## $ patid   : int 1004 1005 1008 1015 1025 1035 1037 1038 1043 …

## $ eventdate : chr "2007-12-25" "2004-08-31" "2002-03-02" …

## $ medcode  : int 351 351 351 351 351 293 277 273 351 257 …

## $ table  : chr "Clinical" "Clinical" "Clinical" "Clinical" …

## $ year   : int 2008 2008 2008 2008 2008 2008 2008 2008 2008 …

Note that in this case extra columns have been added for both year and table, to identify the table the event was found in. Because events were taken from more than one table (Clinical and Referrals), the incident_cases dataframe should be sorted and duplicates removed to ensure that only the first events are kept. The two datasets are then merged to give the dataset from which the denominators and numerators can be calculated. The dplyr package is imported to the namespace when the rEHR package is loaded. This simplifies and accelerates merging operations, using left_join from the dplyr package in the example below, and is an important part of the rEHR workflow:

## All patients are kept (equivalent to merge(all.x = TRUE))

prevalence_dat <- left_join(registered_patients, incident_cases)

## Remove duplicates across clinical and referral tables:

incident_cases %>%

  group_by(patid, year) %>%

  arrange(eventdate) %>%

  distinct() %>%

  ungroup -> incident_cases

Prevalence and incidence can be calculated by the built-in functions prev_terms() and prev_totals(). prev_terms() adds logical columns for membership of incidence and prevalence denominators as well as a column for the contribution of the individual to that year’s followup time. prev_totals() summarises this information to calculate the denominators and numerators for prevalence and incidence, according to the users’ grouping factors. The criteria for membership of the incidence and prevalence numerators and denominators as well as for followup time are shown in Table 1, where event date is the date the event of interest occurs, transfer out date is the date the patient (may have) exited the practice or the database, and year start date is either 1st of Jan (calendar) or 1st of April (financial).

**Table 1:** Definitions of incidence and prevalence terms.

Term	Definition
Incident Numerator	event occurs within year AND transfer out date > event date
Incident Denominator	No events in previous years AND transfer out date > year start date
Prevalent Numerator	event occurs within year AND transfer out date > event date
Prevalent Denominator	transfer out date > year start date
Follow-up	minimum of (year end date, transfer out date, death date)—year start date

An example in the use of these functions is provided below:

prevalence_dat <- prev_terms(prevalence_dat)

totals <- prev_totals(prevalence_dat)

totals$prevalence$year_counts

## Source: local data frame [5 x 4]

##

##   year numerator denominator prevalence

## 1 2008     31   174.6721  17.74754

## 2 2009     35   179.3785  19.51181

## 3 2010     41   183.1403  22.38721

## 4 2011     50   185.4182  26.96607

## 5 2012     55   191.5838  28.70806

totals$incidence$year_counts

## Source: local data frame [5 x 4]

##

##   year numerator denominator incidence

## 1 2008      4   143.9014  2.779680

## 2 2009      3   144.4983  2.076149

## 3 2010      4   142.2806  2.811345

## 4 2011      7   135.5893  5.162648

## 5 2012      5   137.4675  3.637224

Here we see that, in our simulated dataset, we have a diabetes prevalence of 17.7% in 2008 raising to 28.7% in 2012 and an incidence of 2.8% in 2008 increasing to 3.6% in 2012.

## 4 Building cohorts, matching and time-varying covariates

In this section we demonstrate how to convert the longitudinal data from the previous section to a cohort dataset suitable for survival analysis and also illustrate algorithms to match controls to cases and to cut cohort data by time-varying covariates.

One of the most common uses of EHR data in research is to build cohorts for survival analyses. The longitudinal data in the previous section is easily converted to survival cohort format using the build_cohort() function. This returns a dataset with a single row for each patient and includes only patients in the numerator or denominator for whichever cohort type is chosen (either incident or prevalent cohorts). Columns are added for start and end dates and for start and end times as integer differences from the cohort start date. A binary column is added to indicate membership of the case group. All patients with start dates greater than their end dates are removed from the dataset. The diagnosis_start argument is used to include the diagnosis date in the definition of the start dates for the patients. If it is not required for the diagnosis date to be included in the start date definition, this argument can be set to NULL. Here, we will first merge in practice data (i.e. dates for when practices are deemed to be up to standard) and then construct the cohort:

practices <- select_events(db = db, tab = "Practice", convert_dates = TRUE)

prevalence_dat <- left_join(prevalence_dat, practices)

cohort <- build_cohort(prevalence_dat, cohort_type = "prev",

            cohort_start = "2006‐01‐01", cohort_end = "2012‐12‐31"

            , diagnosis_start = "eventdate")

The cohort is now ready for analysis, e.g. with a relatively simple proportional hazards regression model that only includes gender and exposure as predictors:

## Add a logical column for death during cohort

cohort$death <- with(cohort,

         ifelse(!is.null(deathdate) &

              (deathdate > as.Date("2006‐01‐01") &

                deathdate < as.Date("2012‐12‐31")),

             1, 0))

cohort$death[is.na(cohort$death)] <- 0

library(survival)

surv_obj <- with(cohort, Surv(start, end, death))

coxph(surv_obj ~ gender + case, data = cohort)

## Call:

## coxph(formula = surv_obj ~ gender + case, data = cohort)

##

##

##      coef exp(coef) se(coef)   z   p

## gender 0.506   1.659  0.837   0.605 0.55

## case  -0.645   0.524  1.081 -0.597 0.55

##

## Likelihood ratio test = 0.81 on 2 df, p = 0.667 n = 199,

## number of events = 7

### 4.1 Matching

Matching cases to controls is an important pre-analysis step. The rEHR package provides three methods for matching cases to controls:

1. Incidence density matching (IDM)
2. Exact matching
3. Matching on a dummy index date sourced from consultation files

#### 4.1.1 Incidence density matching

This is performed using the get_matches() function. With IDM, controls are selected for a particular case at the time of diagnosis (or other event such as death) from other members of the cohort who, at that time, do not have the diagnosis. The IDM sampling procedure allows the same patient to be selected as a control for more than one case, thus providing a full set controls for each case while still producing unbiased estimates of risk [7; 21]. This also means that the matching procedure can be parallelised to increase computational efficiency.

cohort2 <- build_cohort(prevalence_dat, cohort_type = "incid",

            cohort_start = "2006‐01‐01",

            cohort_end = "2012‐12‐31",

            diagnosis_start = "eventdate")

IDM_controls <- get_matches(cases = filter(cohort2, case == 1),

               control_pool = filter(cohort2, case == 0),

               match_vars = c("gender", "region"),

               n_controls = 4, cores = 1,

               method = "incidence_density",

               diagnosis_date = "eventdate")

In this example matching scenario, 92 controls were matched to 23 cases, which is 4 controls matched to each case.

In all of the matching algorithms, matching is performed by default on categories selected in the match_vars argument. However, more complex matching strategies can also be employed via the extra_conditions argument. You can wrap calls to expressions in dotted brackets to automatically expand them. This is particularly useful when you want to find the value for each individual case. Each case is denoted by CASE, e.g. "start_date <.(CASE$start_date)" will ensure the start date for controls is prior to the start date for the matched case. The following code also selects controls whose birth year (yob) is within 2 years either side of their matched case:

IDM_controls2 <- get_matches(cases = filter(cohort2, case == 1),

              control_pool = filter(cohort2, case == 0),

              match_vars = c("gender", "region"),

              extra_conditions = "yob>=(.(CASE$yob)-2)

              &yob<=(.(CASE$yob)+2)",

              n_controls = 4, cores = 1,

              method = "incidence_density",

              diagnosis_date = "eventdate")

#### 4.1.2 Exact matching

Exact matching only matches controls from the control pool, unlike in IDM matching. Also, matched controls are removed from the control pool after each case has been matched, so each control can be used a maximum of one time. Therefore it is possible to have fewer matched controls for some cases than are requested via the n_controls argument. Because the control pool is being altered for every case, exact matching is not thread safe and so will only run on a single core. The cores and diagnosis_date arguments are ignored when this method is selected.

exact_controls3 <- get_matches(cases = filter(cohort2, case == 1),

             control_pool = filter(cohort2, case == 0),

             match_vars = c("gender", "region"),

             n_controls = 4, cores = 1,

             method = "exact",

             diagnosis_date = "eventdate")

In a small cohort, this can rapidly reduce the control pool, leading to many cases without matches. In this example, 19 out of 23 were matched with mean 3.6 controls matched to every case.

#### 4.1.3 Matching on a dummy index date

A common matching approach is to match on an index date, for example the diagnosis date of the cases or the date followup starts. There are several reasons to match on index date:

1. It ensures cases and controls are followed-up, on average, for the same amount of time. Not including an index date for controls may result in them being, on average, in the cohort for longer than the cases because their cohort start date is not constrained by the index date
2. There is a possible reduction of detection bias, for example if cases are expected to visit their doctors more often because they have more co-morbidities
3. If controls are known to have attended their practice at around the same time as their matched case, it is likely they will experience similar conditions in terms of practice policy and active GPs
4. Patients who, though registered, have no records of contact with the medical system (“Ghost patients”) are excluded

However, the controls will often not have the same index to match on (this is true by definition if the diagnosis date is used). In this situation, it is common to match on a dummy index date which may be a clinical event or interaction in the control’s electronic health record that occurs around the same time as the index date of the case [22; 23]. The match_on_index() function allows for matching on an arbitrary number of categorical match_var variables and on continuous variables via the extra_conditions argument in the same way as the get_matches() function above. In addition, a supplied index date for each case is matched to event dates in a series of consultation files (1 file for each practice), providing a dummy index date for controls of a consultation date within index_diff_limit days of the matched case’s index date.

Note that the consultation files must be in flat-file format, i.e. not as part of the database, but as text (or other filetype, e.g stata dta) files. This is the data format provided by CPRD (“Clinical Practice Research Datalink (CPRD) GOLD”). Although in most situations it is more efficient to process EHR data in SQL databases, as in the earlier functions described here, consultation tables are often very large and searching these for every case in a large cohort would be very slow. By processing consultation files that have been split by practice, it is possible to search for matches a practice at a time which is both efficient and allows for parallel processing to speed the process up still further. For convenience, a function flat_files() is provided that can export a database table to flat files split by practice in a format of their choosing. The match_on_index() function has an import_fn argument to use different file formats (e.g. foreign::read.dta or readstata13::read.dta13 for Stata 12 or Stata 13 file).

consultation_dir <- "~/R/rEHR_testing"

flat_files(db, out_dir = consultation_dir, file_type = "csv")

index_controls <- match_on_index(cases = filter(cohort2, case == 1),

               control_pool = filter(cohort2, case == 0),

               index_var = "eventdate",

               match_vars = c("gender", "region"),

               index_diff_limit = 90,

               consult_path = consultation_dir,

               n_controls = 4,

               import_fn = function(x)

                  convert_dates(read.csv(x)))

# clean up constructed dirs after analysis

unlink(consultation_dir, recursive = TRUE)

This function performs matching that is still more conservative than the previous methods, since it requires matching of patients within the same practice and with consultation dates near the index date. In the test example above, no matched controls were found which is not surprising with a control pool of only 143. In practice this method is only appropriate where there is a control pool of hundreds of thousands or even millions of patients. If too few controls are found, the constraint can be relaxed by setting a higher index_diff_limit. Setting this to an arbitrarily high value effectively means that matching is not done on index date, but just on practice and the other user-specified matching variables. Users may find that this is a more efficient way to perform exact matching than using the get_matches() function. We have used this method to accelerate matching runs with several million controls that previously took days or weeks to minutes or a few hours.

### 4.2 Time-varying covariates

Often, researchers want to cut a survival cohort by time-varying covariates. In this situation, individual patients may run over more than one row in the cohort dataset. For example, a drug exposure may occur after the entry into the cohort and one might be interested in how this might affect the outcome. In this situation, it is useful to have a pre-exposure and post-exposure time period in the dataset.

The cut_tv() function cuts up a dataset based on times supplied for the time-varying covariate. If there is already a variable for the time-varying covariate, you can chose to flip the existing values or increment them. This means the function can be called multiple times to, e.g. deal with drugs starting and stopping and also to model the progression of treatment. Other packages implement similar functions (e.g. the cutLexis function from the Epi package [24]). The cut_tv() function is considerably faster than other cutting methods (particularly on large datasetss), does not require conversion of the dataset to other formats (such as Lexis), can be parallelised on posix compliant machines and is designed to be chained with dplyr workflows using the %>% operator. cut_tv() can deal with the following scenarios:

- **Binary chronic covariates** e.g. The time of diagnosis for a chronic (unresolvable) condition. This requires a single column variable of times from entry in the dataset
- **Binary covariates** e.g. times of starting and stopping medication. This requires more than one column variable in the dataset, one for each start or stop event. The state flips with each new change.
- **Incremental time-varying covariates** e.g. different stages of a condition. This requires a single column variable for each incremental stage
- **Any combination of the above** This is achieved by chaining multiple calls together

One must supply a dataframe, variable names for entry and exit times, the time-varying covariate, the patient id and the constructed variable. Also one supplies the number of processor cores to run the function on and the behaviour of the function if the constructed variable already exists (either to flip from 1-0 or to increment by one). Here we demonstrate the different scenarios with a small sample dataset:

tv_test <- data.frame(id = 1:5, start = rep(0, 5),

        end = c(1000, 689, 1000, 874, 777),

        event = c(0, 1, 0, 1, 1),

        drug_1 = c(NA, NA, NA, 340, 460),

        drug_2 = c(NA, 234, 554, 123, NA),

        drug_3_start = c(110, 110, 111, 109, 110),

        drug_3_stop = c(400, 400, 400, 400, 400),

        stage_1 = c(300, NA, NA, NA, NA),

        stage_2 = c(450, NA, NA, NA, NA))

## Multiple binary chronic covariates:

tv_out1 <- cut_tv(tv_test,

        entry = start,

        exit = end,

        cut_var = drug_1,

        id_var = id,

        tv_name = drug_1_state)

tv_out1 <- cut_tv(tv_out1, start, end, drug_2, id_var = id, drug_2_state)

head(tv_out1)

## Source: local data frame [6 x 12]

##

##  id start  end  event  drug_1 drug_2  drug_3_start drug_3_stop stage_1

## 1 1   0 1000   0   NA   NA      110     400   300

## 2 2   0  233   1   NA   234      110     400   NA

## 3 2  234  689   1   NA   234      110     400   NA

## 4 3   0  553   0   NA   554      111     400   NA

## 5 3  554 1000   0   NA   554      111     400   NA

## 6 4   0  122   1   340   123      109     400   NA

## Variables not shown: stage_2 (dbl), drug_1_state (dbl),

## drug_2_state (dbl)

## Binary covariates:

tv_out3 <- cut_tv(tv_test, start, end, drug_3_start, id_var =

    id, drug_3_state)

tv_out3 <- cut_tv(tv_out3, start, end, drug_3_stop, id_var =

    id, drug_3_state)

head(tv_out3)

## Source: local data frame [6 x 11]

##

##  id start  end event drug_1 drug_2 drug_3_start drug_3_stop stage_1

## 1 1   0  109   0   NA   NA     110     400  300

## 2 1  110  399   0   NA   NA     110     400  300

## 3 1  400  1000   0   NA   NA     110     400  300

## 4 2   0  109   1   NA   234     110     400  NA

## 5 2  110  399   1   NA   234     110     400  NA

## 6 2  400  689   1   NA   234     110     400  NA

## Variables not shown: stage_2 (dbl), drug_3_state (dbl)

## incremental covariates:

inc_1 <- cut_tv(tv_test, start, end, stage_1, id_var = id, disease_stage,

         on_existing= "inc")

inc_1 <- cut_tv(inc_1, start, end, stage_2, id_var = id, disease_stage,

         on_existing= "inc")

head(inc_1)

## Source: local data frame [6 x 11]

##

##  id start  end event drug_1 drug_2 drug_3_start drug_3_stop stage_1

## 1 1   0  299   0   NA   NA     110     400   300

## 2 1 300  449   0   NA    NA     110     400   300

## 3 1 450 1000   0   NA   NA     110     400   300

## 4 2   0  689   1   NA   234     110     400   NA

## 5 3   0 1000   0   NA   554     111     400   NA

## 6 4   0  874   1  340   123     109     400   NA

## Variables not shown: stage_2 (dbl), disease_stage (dbl)

## Chaining combinations of the above using %>%

library(dplyr)

tv_test %>%

  cut_tv(start, end, drug_1, id_var = id, drug_1_state) %>%

  cut_tv(start, end, drug_2, id_var = id, drug_2_state) %>%

  cut_tv(start, end, drug_3_start, id_var = id, drug_3_state) %>%

  cut_tv(start, end, drug_3_stop, id_var = id, drug_3_state) %>%

  cut_tv(start, end, stage_1, id_var = id, disease_stage,

     on_existing = "inc") %>%

  cut_tv(start, end, stage_2, id_var = id, disease_stage,

     on_existing = "inc") %>%

  head

## Source: local data frame [6 x 14]

##

##  id start  end event drug_1 drug_2 drug_3_start  drug_3_stop  stage_1

## 1 1   0  109   0   NA   NA     110     400   300

## 2 1  110  299   0   NA   NA     110     400   300

## 3 1  300  399   0   NA   NA     110     400   300

## 4 1  400  449   0   NA   NA     110     400   300

## 5 1  450 1000   0   NA   NA     110     400   300

## 6 2   0  109   1   NA   234     110     400   NA

## Variables not shown: stage_2 (dbl), drug_1_state (dbl), drug_2_state

## (dbl), drug_3_state (dbl), disease_stage (dbl)

## 5 Accessory functions

In this section we briefly discuss some miscellaneous functions provided in the package.

### 5.1 Clinical code list construction

An important part of EHR analyses is the construction of lists of clinical codes to define conditions, comorbidities and other clinical entities of interest to the study [9]. We have previously described methodologies to construct draft lists of clinical codes from keyword and code searches [19]. The R implementation of this methodology is now part of the rEHR package.

Building draft lists of clinical codes is a two-stage process: First, the search is defined by instantiating an object of class MedicalDefinition, containing the terms to be searched for in the lookup tables. MedicalDefinition objects can be instantiated from terms defined within R or imported from a csv file. The constructor function can be provided with lists of: terms(clinical search terms), codes (clinical codes), tests (test search terms), drugs (drug search terms), drugcodes (drug product codes). Within the individual argument lists, vectors of length > 1 are searched for together (logical AND), in any order. Different vectors in the same list are searched for separately (logical OR). Placing a “-” character at the start of a character vector element excludes that terms from the search. Providing NULL to any of the arguments means that this element will not be searched for. Underscores are treated as spaces. When searching for codes, a range of clinical codes can be searched for by providing two codes separated by a hyphen. e.g “E114-E117z”.

## Example construction of a clinical code list

def <- MedicalDefinition(

  terms = list(

    "peripheral vascular disease", "peripheral gangrene",

    "‐wrong answer", "intermittent claudication",

    "thromboangiitis obliterans", "thromboangiitis obliterans",

    "diabetic peripheral angiopathy",

    c("diabetes", "peripheral angiopathy"), # single AND expression

    c("buerger", "disease presenile_gangrene"),

      "‐excepted", # exclusion

  codes = list("G73"),

  tests = NULL,

  drugs = list("insulin", "diabet", "aspirin")))

Code lists can be defined in a csv file with format as shown in Table 2. These files can then be imported to MedicalDefinition objects using the import_definitions(input_file = "path/to/file.csv") function.

**Table 2:** Example code list definition in csv format.

definition	status	items	
terms	include	peripheral vascular disease	peripheral angiopathy terms disease presenile_gengrene terms
terms	include	peripheral gangrene	
terms	exclude	wrong answer	
terms	include	intermittent claudication	
terms	include	thromboangiitis obliterans	
terms	include	Diabetic peripheral angiopathy	
terms	include	diabetes	
terms	include	buerger	
terms	exclude	excepted	
codes	include	G73	
drugs	include	insulin	
drugs	include	aspirin	

The MedicalDefinition objects are then used to run searches against lookup tables provided with EHRs via the build_definition_lists() function:

## Use fileEncoding = "latin1" to avoid issues with non-ascii characters

medical_table <- read.delim("Lookups/medical.txt", fileEncoding = "latin1", stringsAsFactors = FALSE)

drug_table <- read.delim("Lookups/product.txt", fileEncoding = "latin1", stringsAsFactors = FALSE)

draft_lists <- build_definition_lists(def, medical_table= medical_table, drug_table = drug_table)

### 5.2 Unit conversion

HbA1C tests for glycated haemoglobin are one of the best recorded clinical tests in UK primary care databases, to a large extent because of testing being incentivised under the UK Quality and Outcomes Framework pay-for-performance scheme [20; 25]. However, HbA1C data is not recorded in CPRD consistently. Measurements may have been made in mmol/mol, mmol/L or mg/dL. Also the closely analogous fructosamine test can also be converted into the same units for direct comparison. The CPRD-specific cprd_uniform_hba1c_values() function accepts a single argument of a dataframe in the CPRD “Additional” table form containing only entity types for HbA1C and Fructosamine and converts any HbA1C and fructosamine values to a common mmol/mol scale. Once this conversion has taken place, the function also removes obvious mis-coding errors that are far outside the possible range. A dataframe is returned with an extra column hba1c_score.

### 5.3 Exporting data to stata format

Sometimes researchers may need to share data with others in the same group who may not have R expertise. We have provided the to_stata function to export dataframes to stata dta format. This function compresses a dataframe to reduce file size in the following ways:

1. Date variables (as specified by the date_fields argument) are converted to integer days from 1960-01-01 to avoid compatibility issues between R and Stata. An alternative origin can be set with the origin argument.
2. Fields specified in the integer_fields are converted from numeric to integer.

the stata13 boolean argument indicates whether files should be stored in Stata13 format (Using readstata13::savedta13) or in Stata 12 compatible format (using foreign::write.dta). The former includes a further compression step, similar to the compress command in Stata.

### 5.4 Working with temporary database tables

The size of EHR databases may require keeping intermediate data extractions as database tables, rather than as in-memory R dataframes. For example, extractions of clinical events for a common condition such as diabetes or asthma will require the extraction of millions of rows of data. These may be easily stored as temporary database tables. This is also useful if you are working with a protected database that you only have read-only access to. The rEHR package has a suite of functions to deal with temporary database tables:

- temp_table() is used to construct temporary tables and is illustrated in section 3
- append_to_temp_table() appends rows to a temporary table based on a specified select statement
- to_temp_table() exports a dataframe to a temporary database table
- drop_temp_table() checks if a temporary table exists and then deletes if it does
- drop_all_temp_tables() drops all temporary tables from the database

Note that temporary tables are only associated with the currently open database connection. This means that functions capable of parallel processing (e.g. select_by_year()) can only be used in the single core mode (i.e. set cores = 1) since multicore processes open up multiple parallel connections.

## 6 Setting EHR type

In the final section we discuss the .ehr environment used to define the EHR database being used and how this can be set to work with different databases.

In many of the functions in this package, specific tables and variables in the database need to be accessed. A particular database system, such as CPRD, will have its own schema describing the organisation of the data within it. To simplify the functions in this package, we have opted to include an interface to the database schema in the form of an environment, .ehr, that is accessed by the various analysis functions in order to extract the correct data from the correct place in the database. This is effectively a list of attributes relating to the EHR system being used. For example there is an attribute specifying the patient id variable in the database. By default, a schema environment for CPRD is loaded when the package is loaded via a call to set_CPRD(). We have provided accessor functions to get and set attributes in the .ehr environment. It is preferable to use these accessor functions rather than setting elements directly. A list of all of the attributes is provided by the list_EHR_attributes() function. For example:

list_EHR_attributes()

##  [1] "birth_year"     "cohort"     "date_fields"

##  [4] "ehr_medcode"   "EHR_name"   "event_date"

##  [7] "lookup"      "patient_id"   "practice_id"

## [10] "raw_date_format" "tables"     "year_origin"

The values of individual attributes can be accessed with the get_EHR_attribute() function:

get_EHR_attribute(patient_id) # gives the attribute for patient ids

## [1] "patid"

get_EHR_attribute(date_fields) # fields in the database stored as dates

##    event    entry   last_coll up_to_std first_reg

## "eventdate"   "sysdate"    "lcd"   "uts"  "frd"

## current_reg transfer_out    death

##    "crd"    "tod" "deathdate"

get_EHR_attribute(cohort) # variables used in cohort construction

## $start_criteria

## [1] "crd" "uts"

##

## $end_criteria

## [1] "tod"  "deathdate" "lcd"

Individual attribute values can be set using the set_EHR_Attribute() function:

# set the patient id attribute

set_EHR_attribute(patient_id, value = "PATIENT")

get_EHR_attribute(patient_id)

## [1] "PATIENT"

The default settings can be reverted to using the set_CPRD() function:

set_CPRD()

## Using CPRD settings

get_EHR_attribute(patient_id)

## [1] "patid"

The .ehr environments will allow for the simple definition of interfaces to other EHR systems, via the construction of new setting functions.

## 7 Conclusion

Working with structured EHR data requires a combination of computational and statistical expertise. The rEHR package greatly simplifies and accelerates the extraction and processing of coded data from EHR databases, enabling researchers to spend more time on their analyses, time that would otherwise be consumed with laborious preparation of research-ready data. The workflow is straightforward, amounting to a flat series of function calls rather than a complex set of nested loops, therefore errors are much more easily spotted and fixed. The available functions are summarised in Table 3. The combination of SQL native databases, optimised data manipulation packages and multicore functionality results in a package that runs many times faster than equivalent code.

**Table 3:** Available functions in rEHR.

code file	function	description
codelists	extract_keywords	Function to extract rows from a lookup table based on keywords
	MedicalDefinition	Constructor function for MedicalDefinition class
	import_definitions	Imports definitions to be searched from a csv file into a MedicalDefinition object
	export_definition_search	Exports definition searches to an excel file
	definition_search	This function is used to build new definition lists based on medical definitions
	print.MedicalDefinition	Basic print method for medical definition classes
cohort	build_cohort	Converts a longitudinal data set from e.g. ∖*code*{*prev*_*terms*} to a cohort dataset
	cut_tv	Cuts a survival dataset on a time varying variable
cprd_import	read_zip	Reads a zipped data file to a dataframe
	database	Wrapper for dbConnect
	add_to_database	Adds a series of files to a database
	import_CPRD_data	Imports all selected CPRD data into an sqlite database
cprd_medcodes	patients_per_medcode	Produce a dataset of CPRD medcodes with frequencies of patients in the clinical table
	medcodes_to_read	Translate CPRD medcodes to Read/OXMIS
	read_to_medcodes	Translate Read/Oxmis codes to CPRD medcodes
cprd_patients	patients_in_window	Select patients alive and registered between certain dates
data	clinical_codes	Clinical codes for 17 QOF conditions, smoking and HbA1c
	entity	A sample of 6 clinical tests and meaures used in UK primary care
	product	A sample of 500 medicines used in UK primary care
	repsample_example	An example dataset to demonstrate the repsample function. 2474 theoretical UK general practices
	ehr_def	An example EHR_definition object for defining parameters for simulating EHR data
db_view	head.SQLiteConnection	head for SQLiteConnection object
EHR_definition	define_EHR	Construct an EHR_definition object
	print.EHR_definition	Tools for describing EMR_description objects
ehr_simulation	random_dates	Generates random dates between a start and end day
	surv_sims	Function to simulate survival data
	simulate_ehr_patients	Generate a dataframe of simulated patients with exit dates based on presented comorbidities
	simulate_ehr_practices	Generate a simulated dataframe of primary care practices in the same format as is used in the CPRD
	simulate_ehr_consultations	Generates simulated GP consultation tables
	simulate_ehr_events	Generate simulated events tables
ehr_system	set_CPRD	Sets EHR metadata to CPRD format
	get_EHR_attribute	Return the value of an attribute in the .ehr environment
	set_EHR_attribute	Sets the value of an attribute in the .ehr environment
	list_EHR_attribute	Lists all of the EHR attribute names in .ehr
matching	match_case	Selected controls matching a list of variables from a case
	get_matches	Find matched controls for a set of cases
	match_on_index	Function for performing matching of controls to cases using the consultation files to generate a dummy index date for controls
prevalence	prev_terms	Adds columns enabling one to calculate numerators and denominators for prevalence and incidence
	prev_totals	Calculates the prevalence totals for the output of a data frame of events/patients etc
select_by_year	select_by_year	Runs a series of selects over a year range and collects in a list of dataframes
	build_date_fn	Function to build start/enddate helper fuctions
	qof_years_fn	Helper function providing startdate and enddate for QOF years
	qof_15_month_fn	Helper function providing startdate and enddate for QOF 15 month periods
	standard_years_fn	Helper function providing startdate and enddate for calendar years
select_events	select_events	Extracts from the database
	first_events	Selects the earliest event grouped by patient
	last_events	Selects the latest event grouped by patient
temp_tables	temp_table	Creates a temporary table in the database
	append_to_temp_table	Appends rows to a temporary table
	to_temp_table	Send a dataframe to a temporary table in the database
	drop_temp_table	Checks if a temporary table exists and then deletes if it does
	drop_all_temp_tables	Checks if any temporary tables exist and then deletes all
	temp_location	Sets location of the db temporary store for temporary tables
uniform_units	cprd_uniform_hba1c_values	Standardises HbA1C values to mmol/mol
utils	compress	Compresses a dataframe to make more efficient use of resources
	to_stata	Compresses a dataframe and saves in stata format. Options to save as Stata 12 or 13
	wrap_sql_query	Combines strings and vectors in a sensible way for select queries
	expand_string	Reads strings and expands sections wrapped in dotted parentheses
	convert_dates	Converts date fields from ISO character string format to R Date format
	export_fn	Exports to a variety of formats based on the file type argument
	flat_files	Exports flat files from the database. One file per practice

### 7.1 Limitations and future work

Although rEHR is currently only tested with CPRD data, the .ehr environment system will allow it to be easily linked to other EHR databases. For future versions of the rEHR software we will consider:

- Implementation of the repsample algorithm for representative sampling of practices [26].
- Iterative proportional fitting for matching on population characteristics between different EHR databases [8].
- A robust algorithm for determining smoking status.
- Interfaces to other EHR systems, in particular UK primary care databases such as THIN, QResearch and Research One.
- Uniform units functions for other clinical measurements such as blood pressure, cholesterol and serum creatinine.
- Probabilistic or other matching for patient record linkage.

## Ethics and consent statement

This study is based on data from the Clinical Practice Research Datalink (CPRD) obtained under licence from the UK Medicines and Healthcare products Regulatory Agency. However, the interpretation and conclusions contained in this paper are those of the authors alone. The study was approved by the independent scientific advisory committee (ISAC) for CPRD research (reference number: 16_115R). No further ethics approval was required for the analysis of the data.
